# Generative AI Use and Critical Thinking Dispositions in Higher Education: A Cross-Sample Study of the Sequential Role of Metacognitive Weakness and Epistemic Laziness

**DOI:** 10.3390/jintelligence14070147

**Published:** 2026-07-13

**Authors:** Kağan Kırcaburun

**Affiliations:** Educational Sciences Department, Düzce Üniversitesi, Düzce 81620, Turkey; kircaburunkagan@gmail.com

**Keywords:** artificial intelligence, critical thinking dispositions, metacognitive weakness, epistemic laziness, university students

## Abstract

As generative artificial intelligence (GAI) technologies become increasingly integrated into university students’ academic routines, concerns have emerged regarding their associations with critical thinking dispositions during GAI use. The main objective of the present study was to examine whether metacognitive weakness and epistemic laziness individually and sequentially accounted for the association between GAI tool usage and critical thinking dispositions during GAI use across two distinct student samples. The Turkish-speaking sample consisted of 441 students, whereas the English-speaking sample included 218 students. Participants completed measures assessing GAI tool usage, critical thinking dispositions in GAI use, metacognitive weakness in GAI use, and epistemic laziness. Measurement invariance analyses indicated configural and metric invariance across groups. Path analyses revealed that GAI tool usage had significant indirect associations with lower critical thinking dispositions during GAI use via metacognitive weakness, epistemic laziness, and a sequential indirect pathway involving both constructs. The sequential indirect associations were significant in both the Turkish-speaking sample and the English-speaking sample. Overall, the findings suggest that greater GAI tool usage among university students is associated with lower critical thinking dispositions, with this association statistically carried through metacognitive weakness and epistemic laziness. The consistency of this pattern across two distinct student samples provides preliminary support for a process-oriented interpretation of how GAI use may relate to critical thinking dispositions.

## 1. Introduction

Generative artificial intelligence (GAI) has rapidly evolved from an emerging technology into a routine part of university students’ academic lives. Students increasingly use GAI systems to support learning and academic work (Chan & Hu, 2023; Yurt & Kuşci, 2026). Research in higher education suggests that adoption is widespread but varies across educational settings and students’ prior experiences with the technology (Chan & Hu, 2023; Zhang et al., 2024). Educational research has increasingly turned its attention to how academic thinking changes as learning becomes more closely intertwined with machine-generated responses (Dizon et al., 2026; Gerlich, 2025; Lee et al., 2025).

Critical thinking is central to this discussion because it may be among the cognitive capacities most affected by GAI use (Gerlich, 2025; Zhang et al., 2024). In higher education, critical thinking refers to reflective evaluation and reasoned judgment rather than simply producing acceptable answers (Sosu, 2013). From an intelligence perspective, these abilities support adaptive functioning by helping individuals monitor understanding and revise judgments under uncertainty (Halpern & Dunn, 2021). This becomes particularly important in GAI-assisted learning because generative systems often produce fluent and persuasive outputs that are weakly grounded or inaccurate, creating epistemic risks when students accept them uncritically (Farrokhnia et al., 2024; Hannigan et al., 2024). Accordingly, critical thinking in GAI use should be understood as an active process of evaluating and questioning GAI-generated information before deciding how it should be used (Lee et al., 2025; Yurt & Kuşci, 2026).

Structured uses of GAI may support higher-order thinking by encouraging reflection and active engagement with ideas (X. Chen et al., 2025; Darmawansah et al., 2025). When students are prompted to evaluate and revise GAI-generated outputs rather than accept them uncritically, critical thinking may be maintained or even strengthened (Borge et al., 2024; X. Chen et al., 2025; de la Puente et al., 2024). These findings suggest that the educational value of GAI depends largely on how it is incorporated into learning activities rather than on the technology itself (Lee et al., 2025; Tian & Zhang, 2025).

By contrast, less structured uses of GAI may be associated with lower cognitive engagement and greater reliance on external support when the technology substitutes for students’ own reasoning (Bastani et al., 2025; Darvishi et al., 2024; Lee et al., 2025). Together, these findings suggest that differences in educational outcomes may be better understood in terms of the cognitive and metacognitive processes involved during GAI use, providing the basis for the theoretical framework developed below (Dizon et al., 2026; Fan et al., 2025).

Cognitive offloading provides a useful framework for understanding these processes. Cognitive offloading refers to relying on external tools to reduce internal cognitive demands (Risko & Gilbert, 2016). This process may support learning when it creates space for more complex thinking, but it may also become problematic when learners reduce engagement with processes they would otherwise need to develop themselves. Earlier work on the Google effect showed that access to digital information may influence how individuals distribute reliance between internal memory and external resources (Sparrow et al., 2011). GAI may extend this process because it goes beyond storing or retrieving information and can provide explanations and generate responses that resemble reasoning. As a result, students may increasingly rely on GAI during activities that involve interpretation and evaluation (Gerlich, 2025; Lee et al., 2025). Related work on student agency suggests that learners may benefit from GAI support without fully developing the underlying strategies required to perform independently (Bastani et al., 2025; Darvishi et al., 2024).

Within this perspective, metacognition may represent an important mechanism. Metacognition refers to monitoring and regulating one’s own thinking (Flavell, 1979; Veenman et al., 2006). In GAI-assisted learning, metacognitive weakness may emerge when students do not actively evaluate whether they understand or can justify GAI-generated information (Yurt & Kuşci, 2026). Because GAI outputs are often fluent and immediately available, students may interpret ease of access as evidence of understanding even when deeper evaluation has not occurred (Fan et al., 2025). Emerging work on metacognitive offloading similarly suggests that learners may rely on GAI during self-regulatory processes that would otherwise require active reflection (Dizon et al., 2026; Fan et al., 2025). From this perspective, metacognitive weakness may represent one pathway through which frequent GAI use becomes associated with changes in learning processes (Yurt & Kuşci, 2026).

A closely related but conceptually distinct process is epistemic laziness. Whereas metacognitive weakness concerns monitoring one’s own thinking, epistemic laziness refers to reduced willingness to invest effort in evaluating knowledge claims (Yurt & Kuşci, 2026). In GAI-assisted contexts, this may involve accepting GAI-generated information with limited scrutiny or relying on convenient responses instead of further evaluation (Gerlich, 2025; Yurt & Kuşci, 2026). This perspective is consistent with theories of epistemic vigilance, which emphasize that individuals actively evaluate the reliability of communicated information (Sperber et al., 2010). It also aligns with evidence suggesting that vulnerability to misinformation may reflect reduced analytical engagement rather than bias alone (Pennycook & Rand, 2019). Because GAI systems often generate fluent and persuasive outputs, students may become less likely to question information when evaluation is replaced by convenience (Hannigan et al., 2024; Yurt & Kuşci, 2026).

Distinguishing metacognitive weakness from epistemic laziness may be theoretically important because these processes could be related rather than independent. Dual-process perspectives suggest a distinction between monitoring one’s own thinking and deciding whether to invest effort in evaluating information (Pennycook & Rand, 2019; Veenman et al., 2006). Related work on epistemic motivation proposes that lower investment in evaluation may emerge when self-regulatory processes become less active (Kruglanski et al., 2010; Yurt & Kuşci, 2026). Meta-reasoning frameworks similarly suggest that monitoring processes provide the basis for control decisions, such that judgments about one’s own understanding determine whether additional cognitive effort is initiated (Ackerman & Thompson, 2017). When monitoring is weak, individuals may fail to recognize that further evaluation is needed, making effortful scrutiny of information less likely (Ackerman & Thompson, 2017; Fan et al., 2025). Accordingly, metacognitive weakness was specified as preceding epistemic laziness in the proposed model rather than treating the two constructs as parallel processes.

In GAI contexts, students who rely on generated outputs without reflecting on their own understanding may become less likely to notice uncertainty and engage in further evaluation. As a result, lower engagement with understanding may coincide with lower engagement in verification and independent judgment (Dizon et al., 2026; Yurt & Kuşci, 2026). From this perspective, changes in critical thinking may be associated less with how often students use GAI and more with the cognitive processes accompanying that use (Yurt & Kuşci, 2026). This interpretation aligns with recent work suggesting that GAI dependence may relate to lower critical engagement through mechanisms such as reduced effort and overreliance (Gerlich, 2025; Tian & Zhang, 2025). It also supports the broader view that GAI may shift critical thinking toward evaluating and managing information rather than generating it directly (Lee et al., 2025).

Prior empirical work provides an important starting point for this model but leaves several questions unresolved. Yurt and Kuşci (2026) reported that GAI tool usage was negatively associated with critical thinking dispositions during GAI use and that this relationship was indirectly linked to both metacognitive weakness and epistemic laziness among university students. These findings move beyond general discussions of whether GAI is beneficial or harmful and suggest that changes in GAI-related critical thinking may be associated with underlying psychological processes (Acosta-Enriquez et al., 2025; Tian & Zhang, 2025; Yurt & Kuşci, 2026). Nevertheless, two issues remain open. First, treating metacognitive weakness and epistemic laziness as parallel mediators leaves unanswered whether they may reflect a sequential process.

Second, most evidence on GAI use and higher-order cognition has been generated within single educational contexts (Acosta-Enriquez et al., 2025; Tian & Zhang, 2025). Whether these mechanisms operate similarly across different educational environments remains unclear. Comparing Turkish-speaking and English-speaking university students may therefore provide a useful opportunity to examine whether the proposed pattern is observed across samples that differ in language and educational background. If the proposed sequence reflects a broader cognitive process, similar patterns may emerge across groups despite contextual variation (Acosta-Enriquez et al., 2025). Examining these questions may contribute to a more context-sensitive understanding of how GAI-assisted learning relates to critical thinking.

Therefore, the main objective of the present study was to examine whether metacognitive weakness and epistemic laziness sequentially account for the association between GAI tool usage and critical thinking dispositions during GAI use across Turkish-speaking and English-speaking university students. Drawing on cognitive offloading, metacognitive perspectives, and recent work on GAI-assisted learning, the study evaluates a process-oriented model in which greater GAI tool usage is indirectly associated with lower critical thinking dispositions through metacognitive weakness, epistemic laziness, and their sequential association (Risko & Gilbert, 2016; Sperber et al., 2010; Yurt & Kuşci, 2026). Gender, age, and GAI use intensity were controlled for in the analyses because prior findings indicate that these variables may be associated with patterns of GAI engagement and related outcomes (Kırcaburun, 2026; Kırcaburun & Özdemir, 2026; Yurt & Kuşci, 2026).

**Figure 1:**
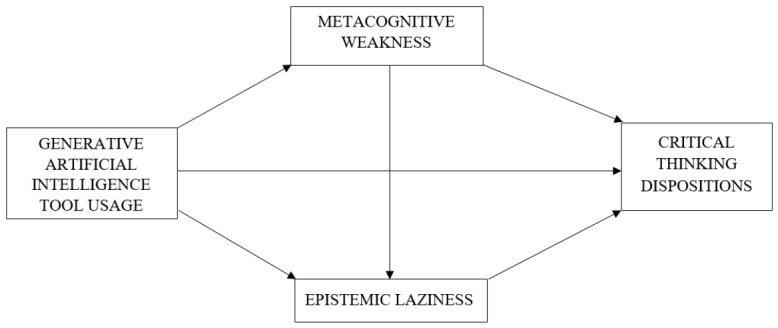
Hypothesized model.

Accordingly, the study addressed the following research question: Do metacognitive weakness and epistemic laziness individually and sequentially account for the association between GAI tool usage and critical thinking dispositions during GAI use, and is this pattern consistent across Turkish-speaking and English-speaking university students? Based on the theoretical framework outlined above, the following hypotheses were tested (see Figure 1). It was hypothesized that GAI tool usage would be indirectly associated with lower critical thinking dispositions during GAI use through metacognitive weakness (H1) and through epistemic laziness (H2). It was further hypothesized that GAI tool usage would be indirectly associated with lower critical thinking dispositions during GAI use through the sequential pathway linking metacognitive weakness and epistemic laziness (H3). Finally, it was hypothesized that this indirect pattern would be observed consistently in both the Turkish-speaking and English-speaking samples (H4).

The study offers three main contributions. Empirically, it extends previous findings by testing metacognitive weakness and epistemic laziness as sequential rather than parallel processes and by examining whether this pattern replicates across two distinct student samples. Methodologically, it evaluates the cross-language equivalence of the measurement instruments through measurement invariance testing and estimates indirect associations using bias-corrected bootstrap procedures. Practically, the findings may inform GAI literacy initiatives and instructional strategies aimed at supporting students’ reflective engagement and critical thinking during GAI-assisted learning.

## 2. Methods

### 2.1. Participants and Procedure

The study employed a quantitative research approach with a cross-sectional, correlational design. This approach was considered appropriate because the aim of the study was to examine the direction and strength of associations among naturally occurring individual difference variables across two samples, rather than to manipulate GAI use experimentally. In terms of scope, the study focused on university students who actively use GAI tools, and the findings are therefore limited to the associations observed within this population.

The study consisted of two separate samples. The Turkish sample was composed of undergraduate students enrolled in the faculty of education at a public university located in northwestern Türkiye. Prior experience with generative artificial intelligence (GAI) tools was defined as an inclusion criterion for participation in the Turkish sample. Data from the Turkish sample were collected through paper-and-pencil questionnaires administered at the beginning of class sessions. The English-speaking sample was recruited through Prolific from a participant pool containing more than 24,000 eligible users. For the Prolific sample, eligibility criteria included currently being a university student, being a native English speaker, and being an active user of GAI technologies (e.g., ChatGPT, Gemini, Grok). A target sample size of 220 participants was specified for the Prolific recruitment process. A total of 222 participants entered the Prolific completion code; however, four participants did not complete the Google Forms questionnaire. Additionally, 14 participants returned the study prior to participation and one participant timed out. Participants were compensated at an hourly rate equivalent to £6. The survey required a median completion time of 4 min and 53 s. Previous methodological investigations have shown that Prolific generally provides high-quality participant samples characterized by reliable responses, substantial demographic variability, and consistent reproducibility of results across studies (Palan & Schitter, 2018; Peer et al., 2022).

The Turkish sample consisted of 441 participants, of whom 81.9% were women, whereas the English-speaking sample included 218 participants, with women comprising 55.5% of the sample. Participants in the Turkish sample ranged in age from 18 to 30 years (*M* = 20.39, *SD* = 1.71), while participants in the English-speaking sample ranged from 18 to 55 years (*M* = 27.78, *SD* = 8.67). All participants in the Turkish sample were undergraduate students. In contrast, the English-speaking sample consisted of 63.8% undergraduate students, 21.1% master’s students, and 7.3% doctoral students. Regarding primary purposes of GAI use, 52.8% of the Turkish participants reported using GAI mainly for educational purposes and 42.6% for general knowledge acquisition. In the English-speaking sample, 77.1% reported educational use and 19.3% reported general knowledge purposes. In terms of weekly GAI usage duration, the majority of Turkish participants reported using GAI for less than two hours per week (73.1%), whereas the most common usage category in the English-speaking sample was between two and five hours per week (35.8%).

Among the English-speaking sample, 38.5% of the participants were from the United States, 28.0% were from the United Kingdom, 26.1% were from Canada, and 7.3% were from other English-speaking countries. Consequently, the two samples differ substantially in age, educational level, gender composition, and recruitment mode. These differences should be taken into account when interpreting cross-sample comparisons, because they may influence both observed score levels and the estimated associations among the study variables. For this reason, the two samples were analyzed separately, demographic variables were included as covariates in the models, and measurement invariance was formally tested before any cross-sample interpretation. Accordingly, cross-sample findings were interpreted in terms of the consistency of the overall pattern of associations rather than as direct quantitative comparisons between the groups. Detailed demographic statistics are presented in Table 1.

Sample sizes exceeding 200 participants are generally regarded as sufficient for obtaining stable parameter estimates and reliable model fit indices (Byrne, 2010; Kline, 2016). The sample sizes of both the Turkish-speaking and English-speaking groups were considered adequate for the conducted analyses, including confirmatory factor analyses, measurement invariance testing, and path analysis. Ethical approval was obtained from the university ethics committee of the research team (Decision no: 2026/84), and all procedures were conducted in accordance with the principles of the Declaration of Helsinki.

### 2.2. Measures

Variables: Participants initially completed a set of items assessing demographic information and patterns of GAI use. For the Turkish-speaking sample, these variables included gender, age, GAI use purpose, and weekly GAI use duration. For the English-speaking sample, the assessed variables included gender, age, country of residence, current educational status, GAI use purpose, and weekly GAI use duration. Participants then completed the Critical Thinking in AI Usage Scale, AI Tool Usage Scale, Metacognitive Weakness in AI Use Scale, and Epistemic Laziness Scale. Both the original Turkish forms and the English versions of the scales were developed by Yurt and Kuşci (2026), with reference to the generative AI systems they actively use, following the procedure adopted in the original scale development study (Yurt & Kuşci, 2026). Accordingly, references to AI in the scale items were interpreted in the context of GAI use. The constructs assessed by these instruments, and the hypothesized associations among them, are summarized in Figure 1. All four scales were retained from the original development study because they were developed simultaneously in Turkish and English by the same authors (Yurt & Kuşci, 2026), which ensured conceptual and linguistic correspondence between the two forms administered in the present samples. The continued use of these scales was further supported in the present study through confirmatory factor analyses, reliability estimates, and measurement invariance testing, which are reported below.

Critical Thinking in AI Usage Scale (CTAIUS): The CTAIUS was originally developed by Yurt and Kuşci (2026) to assess individuals’ reflective and evaluative critical thinking dispositions when interacting with generative artificial intelligence (GAI) systems. The scale was theoretically grounded in Sosu’s (2013) conceptualization of critical thinking disposition, particularly the dimensions of Critical Openness and Reflective Scepticism. In GAI contexts, these dimensions refer to individuals’ tendency to critically evaluate GAI-generated information, question its reliability, reflect on potential consequences, and engage in independent reasoning during GAI-assisted interactions. The original scale consisted of 11 items (“Even if the information presented by AI contradicts my thoughts, I evaluate it by questioning.”) rated on a five-point Likert scale ranging from 1 (Never) to 5 (Always), with higher scores indicating stronger critical thinking dispositions in GAI usage contexts. In the original study, the authors reported that the two theoretically proposed dimensions were highly correlated and therefore supported a unidimensional structure for the scale (Yurt & Kuşci, 2026). In the present study, all 11 original items were retained and used in the analyses (see Supplementary File for the rationale and confirmatory factor loadings). It should be emphasized that the CTAIUS is a self-report dispositional measure. It assesses individuals’ typical tendencies toward evaluating and questioning GAI-generated information rather than their performance-based critical thinking skills.

Confirmatory factor analyses (CFA) were conducted separately for both samples for all assessment tools used in the study (see Supplementary Table S1). Average variance extracted (AVE), composite reliability (CR), McDonald’s omega, and Cronbach’s alpha values indicated robust psychometric properties across the measures. Measurement invariance analyses were further conducted to evaluate the cross-group equivalence of the assessment tools across the Turkish-speaking and English-speaking samples. Detailed psychometric results are presented in Table 2, Table 3 and Table 4.

AI Tool Usage Scale (AITUS): The AITUS was originally developed by Yurt and Kuşci (2026) to evaluate the extent to which individuals incorporate GAI technologies into different areas of everyday life. The instrument assesses the frequency and intensity of GAI-related activities across academic, professional, and personal contexts. The scale includes nine items (e.g., “I use AI-powered tools for my academic/professional tasks.”) assessed on a five-point Likert-type scale ranging from 1 (Never) to 5 (Always). Elevated scores reflect more extensive and intensive integration of GAI tools into participants’ routines, indicating broader reliance on GAI technologies across multiple domains of daily functioning.

Metacognitive Weakness in AI Use Scale (MWAIUS): The MWAIUS was originally developed by Yurt and Kuşci (2026) to assess deficiencies in individuals’ metacognitive awareness during interactions with GAI systems. Metacognitive weakness refers to difficulties in effectively monitoring, evaluating, and regulating one’s own cognitive processes while relying on GAI-generated information and recommendations (Yurt & Kuşci, 2026). The scale captures tendencies such as insufficient self-monitoring, overreliance on GAI outputs, reduced evaluation of understanding accuracy, and prioritization of rapid responses over careful cognitive assessment during GAI-assisted tasks. The instrument consists of five items (e.g., “I neglect to check whether I fully understand AI outputs when using them.”) rated on a five-point Likert scale ranging from 1 (Never) to 5 (Always). Higher scores indicate greater metacognitive weaknesses during GAI usage. The MWAIUS was deliberately designed as a deficit-oriented measure. It operationalizes the absence or insufficiency of monitoring and regulation during GAI use, rather than the full continuum of metacognitive functioning, because the construct of interest in the original development study and in the present study was metacognitive weakness as a potential risk factor in GAI-assisted learning (Yurt & Kuşci, 2026). Accordingly, low scores on the scale indicate the relative absence of these weaknesses rather than high levels of adaptive metacognition, and this measurement property is acknowledged as a limitation in the Discussion section.

Epistemic Laziness Scale (ELS): The ELS was originally developed by Yurt and Kuşci (2026) to assess individuals’ tendency to minimize cognitive effort while obtaining, processing, and evaluating information in the context of generative artificial intelligence (GAI) use. Epistemic laziness refers to a preference for cognitively effortless information processing, characterized by reduced analytical engagement, limited independent evaluation, and a tendency to rely on GAI-generated outputs without sufficient reflection or critical scrutiny (Yurt & Kuşci, 2026). The construct reflects avoidance of effortful reasoning processes and overdependence on rapid, convenient, and externally generated information during GAI-assisted tasks. The scale consists of five items (e.g., “I accept information from AI without questioning it.”) rated on a five-point Likert scale ranging from 1 (Never) to 5 (Always). Higher scores indicate greater epistemic laziness during GAI use, reflecting lower levels of analytical effort, independent reasoning, and critical evaluation of GAI-generated information.

### 2.3. Data Analytic Strategy

All statistical analyses were conducted using IBM SPSS Statistics 26 and AMOS 24. Prior to the analyses, the dataset was screened for missing, inconsistent, and out-of-range values. No missing data were identified. Normality assumptions were evaluated using skewness and kurtosis values prior to the main analyses. Initially, descriptive statistics and Pearson correlation coefficients were computed to examine the distributions and bivariate associations among the study variables. In interpreting the magnitude of the correlations, coefficients of approximately 0.10, 0.30, and 0.50 were considered to represent small, medium, and large associations, respectively (Cohen, 1988). Confirmatory factor analyses (CFA) were performed to examine the construct validity of all multi-item measures used in the study, including the CTAIUS, AITUS, MWAIUS, and ELS. Model fit was evaluated using multiple goodness-of-fit indicators, including the chi-square statistic (χ^2^/df), Comparative Fit Index (CFI), Goodness-of-Fit Index (GFI), Root Mean Square Error of Approximation (RMSEA), and Standardized Root Mean Square Residual (SRMR). Recommended cut-off values proposed in the structural equation modeling literature were considered when evaluating model adequacy (Byrne, 2010; Hu & Bentler, 1999).

Independent-samples *t*-tests were conducted to examine potential differences between the Turkish-speaking and English-speaking samples across the study variables. Additional analyses were conducted within the English-speaking sample to examine potential differences based on country of residence. Country groups (United States, United Kingdom, and Canada) were dummy-coded and included in the analyses to evaluate possible group-based variations across the study variables. To examine the cross-sample equivalence of the measurement instruments, measurement invariance analyses were conducted across the Turkish-speaking and English-speaking samples. Configural, metric, and scalar invariance models were tested sequentially. Measurement invariance was primarily evaluated based on changes in CFI values, as ΔCFI has been suggested to be relatively robust to sample size and model complexity (Cheung & Rensvold, 2002). In addition, changes in Tucker–Lewis index (TLI) and RMSEA values were examined as supplementary indicators during the invariance analyses. ΔCFI and ΔTLI values below 0.01 and ΔRMSEA values below 0.015 were interpreted as evidence of acceptable invariance (F. F. Chen, 2007).

Tolerance and variance inflation factor (VIF) values were examined to assess potential multicollinearity among the predictor variables. Following the preliminary analyses, path analysis was conducted to examine the direct and indirect relationships among GAI tool usage, metacognitive weakness, epistemic laziness, and critical thinking dispositions in GAI usage. The hypothesized structural relations were examined using observed composite scores in a path-analytic framework. This parsimonious approach was selected because the primary aim of the study was to test the proposed indirect association pattern among the focal constructs across two samples. Indirect effects were tested using bias-corrected bootstrap analyses with 10,000 resampling iterations and 95% confidence intervals.

## 3. Results

### 3.1. Descriptive Statistics

As presented in Table 2 (values to the left of the comma represent the Turkish-speaking sample, whereas values to the right represent the English-speaking sample), all study variables demonstrated acceptable reliability and convergent validity across both samples, with Cronbach’s alpha (0.74–0.90), McDonald’s omega (0.74–0.90), composite reliability (0.73–0.90), and average variance extracted (0.35–0.51) values generally meeting recommended thresholds (Lam, 2012). Although several AVE values were below the recommended 0.50 threshold, the composite reliability coefficients exceeded the recommended level, suggesting that the constructs demonstrated acceptable convergent validity despite the relatively low AVE values (Fornell & Larcker, 1981). Skewness (−0.82 to 0.56) and kurtosis (−0.19 to 2.12) values also indicated acceptable univariate normality for all variables (Kline, 2016). Correlation analyses revealed that generative artificial intelligence (GAI) tool usage was negatively associated with critical thinking dispositions (*r* = −0.16 to −0.20, *p* < .01) and positively associated with metacognitive weakness (*r* = 0.33 to 0.38, *p* < .001) and epistemic laziness (*r* = 0.40 to 0.46, *p* < .001). In contrast, critical thinking dispositions demonstrated moderate negative associations with metacognitive weakness (*r* = −0.38 to −0.50, *p* < .001) and epistemic laziness (*r* = −0.51 to −0.52, *p* < .001). Furthermore, metacognitive weakness and epistemic laziness were positively correlated across both samples (*r* = 0.58 to 0.72, *p* < .001).

Regarding demographic and usage-related variables, age demonstrated a small positive association with GAI use in the English-speaking sample (*r* = 0.23, *p* < .01), whereas its correlations with the remaining study variables were generally weak and non-significant across both groups. Gender was negatively associated with critical thinking dispositions in the English-speaking sample (*r* = −0.18, *p* < .01) and positively associated with metacognitive weakness in the Turkish-speaking sample (*r* = 0.10, *p* < .05) and epistemic laziness in the English-speaking sample (*r* = 0.18, *p* < .01). Weekly GAI use duration was non-significantly associated with critical thinking dispositions (*r* = −0.01 to −0.06, *p* > .05) and positively associated with GAI use (*r* = 0.32 to 0.59, *p* < .001), metacognitive weakness (*r* = 0.11 to 0.18, *p* < .05), and epistemic laziness (*r* = 0.22 to 0.24, *p* < .001) across the samples.

Finally, independent-samples *t*-tests indicated that the Turkish-speaking sample demonstrated lower observed scores for critical thinking dispositions (*t*[657] = −8.17, *p* < .001) and higher observed scores for metacognitive weakness (*t*[657] = 4.18, *p* < .001) and epistemic laziness (*t*[657] = 2.21, *p* < .05) than the English-speaking sample. Because scalar invariance was not supported across the groups, these mean-level differences should be interpreted as descriptive observed-score differences rather than as evidence of true latent mean differences. Because item intercepts were not equivalent across the groups, the same latent standing on a construct may correspond to different observed item scores in the two samples. Observed mean differences may therefore partly reflect differences in how the items function across the two language groups rather than genuine differences in the underlying constructs. Additional analyses using dummy-coded country variables within the English-speaking sample revealed that participants from the United States reported lower levels of GAI use (*t*[657] = −2.71, *p* < .01) and metacognitive weakness (*t*[657] = −2.23, *p* < .05), whereas participants from the United Kingdom demonstrated higher levels of GAI use (*t*[657] = 2.50, *p* < .05). Consequently, country groups (United States, United Kingdom, and Canada) were dummy coded and included in the analyses as control variables to account for potential country-based variations across the study variables within the English-speaking sample.

### 3.2. Measurement Invariance

As presented in Table 4, measurement invariance analyses indicated that configural and metric invariance were supported for all scales across the Turkish-speaking and English-speaking samples. The configural models demonstrated acceptable fit indices across all measures (CFI = 0.928–0.993, RMSEA = 0.033–0.061), supporting similarity in factor structures across groups. Metric invariance analyses further indicated acceptable cross-group equivalence of factor loadings, as changes in CFI values remained below the recommended threshold of 0.01 for all scales (ΔCFI = 0.000 to 0.007). In contrast, scalar invariance was not supported, as the scalar models produced substantial deteriorations in model fit (ΔCFI = −0.023 to −0.061; ΔRMSEA = 0.015 to 0.052). Overall, these findings indicate that the scales demonstrated comparable factorial structures and factor loadings across different groups, supporting the suitability of cross-group path analyses and correlational interpretations. However, the lack of scalar invariance suggests that mean-level comparisons, including independent-samples *t*-test findings, should be interpreted with caution due to potential differences in item intercepts.

### 3.3. Model Testing

Prior to the path analyses, variance inflation factor (VIF) and tolerance values were examined to assess potential multicollinearity among the predictor variables. For the Turkish-speaking sample, VIF values ranged between 1.22 and 1.61, whereas tolerance values ranged from 0.62 to 0.82. In the English-speaking sample, VIF values varied between 1.28 and 2.29, while tolerance values ranged between 0.44 and 0.78. These findings indicated no serious multicollinearity concerns across the predictor variables.

For the Turkish-speaking sample, the proposed path model demonstrated a good fit to the data (χ^2^ = 12.37, df = 6, *p* = .05, RMSEA = 0.05, 90% CI [0.00, 0.09], SRMR = 0.03, CFI = 0.99, GFI = 0.99). Gender, age, and weekly GAI use duration were included in the model as control variables. The predictors accounted for 11% of the variance in metacognitive weakness, 38% of the variance in epistemic laziness, and 29% of the variance in critical thinking dispositions in GAI usage. For the English-speaking sample, the model also demonstrated acceptable fit indices (χ^2^ = 20.12, df = 12, *p* = .07, RMSEA = 0.06, 90% CI [0.00, 0.10], SRMR = 0.03, CFI = 0.99, GFI = 0.98). In addition to gender, age, and weekly GAI use duration, country of residence variables (United States, United Kingdom, and Canada) were dummy-coded and included as control variables in the analyses. The model accounted for 14% of the variance in metacognitive weakness, 56% of the variance in epistemic laziness, and 33% of the variance in critical thinking dispositions in GAI usage.

**Figure 2:**
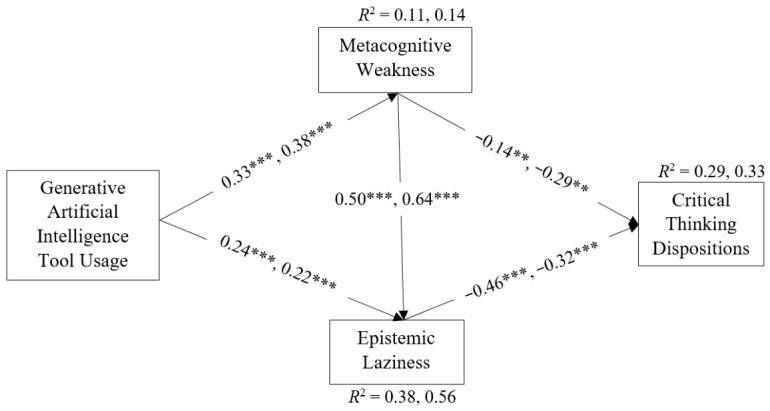
Final model of the significant path coefficients. Note. All variables are included in the model as observed variables and represented as rectangles in the figure. In the reported coefficients, values to the left of the comma represent the Turkish-speaking sample, whereas values to the right represent the English-speaking sample. In both groups, gender, age, and weekly GAI use duration were included in the model as control variables. Additionally, country of residence variables (United States, United Kingdom, and Canada) were dummy-coded and controlled for within the English-speaking sample. For the sake of visual clarity, these control paths, as well as the non-significant direct association between GAI use and critical thinking dispositions, were omitted from Figure 2. Detailed results regarding these variables are presented in Table 5. ** *p* < .01, *** *p* < .001.

Detailed path coefficients are presented in Table 5 and Figure 2. The direct association between GAI use and critical thinking dispositions was non-significant in both samples, indicating a pattern consistent with indirect-only statistical associations through metacognitive weakness and epistemic laziness. In the Turkish-speaking sample, significant indirect effects were identified through metacognitive weakness (*β* = −0.05, *p* < .05; 95% CI [−0.07, −0.01]), epistemic laziness (*β* = −0.11, *p* < .001; 95% CI [−0.14, −0.06]), and the sequential mediation pathway involving both metacognitive weakness and epistemic laziness (*β* = −0.08, *p* < .001; 95% CI [−0.10, −0.05]). Similarly, in the English-speaking sample, the indirect effects through metacognitive weakness (*β* = −0.11, *p* < .05; 95% CI [−0.14, −0.09]), epistemic laziness (*β* = −0.07, *p* < .001; 95% CI [−0.13, −0.03]), and the sequential mediation pathway (*β* = −0.08, *p* < .001; 95% CI [−0.15, −0.04]) were all statistically significant.

## 4. Discussion

The present study examined whether metacognitive weakness and epistemic laziness statistically accounted for the association between GAI tool usage and critical thinking dispositions during GAI use across Turkish-speaking and English-speaking university student samples. The findings showed that GAI tool usage was negatively associated with critical thinking dispositions at the bivariate level, although its direct association became non-significant after metacognitive weakness and epistemic laziness were included in the path models. Both variables were associated with significant indirect pathways in both samples, and the sequential pathway linking GAI tool usage, metacognitive weakness, epistemic laziness, and critical thinking dispositions was also supported across groups. Overall, the findings suggest that the relationship between GAI use and critical thinking dispositions may be better understood through differences in self-monitoring and epistemic engagement rather than as a simple direct association with tool use (Fan et al., 2025; Yurt & Kuşci, 2026). Given the self-report and dispositional nature of the measures, the tested model should be interpreted as describing associations among students’ perceptions of their own GAI-related thinking rather than as a validated account of performance-based critical thinking processes.

The non-significant direct association is theoretically informative because it suggests that GAI use itself may not be uniformly associated with critical thinking outcomes (Tian & Zhang, 2025; Zhao et al., 2025). Recent evidence increasingly indicates that the educational implications of GAI depend less on use frequency and more on how students engage with these systems (Adewumi et al., 2025). Consistent with this view, Si et al. (2026) found that GAI usage frequency predicted perceived efficiency gains but not perceived cognitive gains among Chinese university students, and that deeper technical engagement with GAI paradoxically predicted greater relinquishment of cognitive autonomy, suggesting that how students offload cognitive work matters more than how much they use these systems. Meta-analytic findings suggest that GAI may support higher-order thinking, particularly among learners with stronger self-regulated learning skills (Zhao et al., 2025). Related work in GAI-supported learning similarly emphasizes the role of reflective engagement with GAI systems (Yang et al., 2026). The present findings align with this perspective by suggesting that lower critical thinking dispositions during GAI use may be associated with lower metacognitive engagement and reduced epistemic effort (Gerlich, 2025; Yurt & Kuşci, 2026). Accordingly, the findings suggest that the educational implications of GAI may depend partly on how actively students engage with evaluating information and reflecting on their own understanding (Adewumi et al., 2025; Morais et al., 2026).

The indirect pathway through metacognitive weakness highlights the potential role of self-monitoring during GAI-assisted learning. Because GAI systems often generate fluent and readily available responses, students may develop a sense of understanding before evaluating whether they actually understand or could apply the information independently (Fan et al., 2025). From this perspective, metacognitive weakness may reflect reduced engagement in monitoring one’s own learning while relying on GAI outputs. This interpretation aligns with recent work on metacognitive offloading, suggesting that learners may increasingly rely on GAI during regulatory processes that would otherwise require active reflection (Dizon et al., 2026; Fan et al., 2025). This may be particularly relevant for critical thinking because higher-order cognition involves not only access to information but also the ability to monitor understanding and revise judgments when needed (Halpern & Dunn, 2021). An important issue concerns how this pathway was measured. Because the MWAIUS assesses only deficits in metacognitive functioning, the present findings reflect associations within the lower end of the metacognitive continuum rather than metacognitive functioning across its full range. Whether adaptive metacognitive functioning shows a similar or different pattern of associations with critical thinking dispositions cannot be determined from the present data. This issue is discussed further in the limitations.

The indirect pathway through epistemic laziness further suggests that critical thinking dispositions during GAI use may depend partly on students’ willingness to evaluate knowledge claims. Epistemic laziness was negatively associated with critical thinking dispositions in both samples and accounted for part of the association between GAI use and critical thinking dispositions. This pattern aligns with epistemic vigilance perspectives, which emphasize evaluating information rather than accepting it without reflection (Sperber et al., 2010). It is also consistent with evidence suggesting that lower analytical engagement may contribute to vulnerability to questionable information (Pennycook & Rand, 2019). In GAI-assisted contexts, this issue may be particularly relevant because generated responses can appear convincing even when they are incomplete or insufficiently grounded. From this perspective, repeated reliance on convenient outputs may be associated with lower engagement in evaluating information during learning (Adewumi et al., 2025; Bastani et al., 2025; Hannigan et al., 2024).

The sequential indirect pathway may represent an important extension of previous work. Yurt and Kuşci (2026) reported that metacognitive weakness and epistemic laziness mediated the association between GAI tool usage and critical thinking dispositions, but these mechanisms were examined as parallel processes. The present findings suggest that they may also operate as part of a sequential pattern. One possible interpretation is that students who rely on GAI without reflecting on their own understanding may become less likely to notice uncertainty or recognize when further evaluation is needed (Dizon et al., 2026; Fan et al., 2025). Lower engagement in self-monitoring may then coincide with lower willingness to evaluate information more critically, because students who do not actively reflect on their understanding may also engage less in verification and independent judgment (Yurt & Kuşci, 2026). From this perspective, lower critical thinking dispositions may be associated with a sequence in which metacognitive engagement decreases and epistemic effort becomes less active.

This interpretation should not be understood as evidence of temporal ordering because the data are cross-sectional. However, a similar indirect pattern was observed in both Turkish-speaking and English-speaking samples, which may support the relevance of the proposed process across different educational contexts. Although the samples differed in several demographic and contextual characteristics, the general pattern of associations remained similar. Measurement invariance analyses further supported configural and metric invariance, suggesting comparable factor structures and factor loadings across groups. At the same time, scalar invariance was not supported, and the findings should therefore not be interpreted as evidence of equivalent latent levels or broad cultural generalizability. In practical terms, the absence of scalar invariance means that the reported between-group differences cannot be attributed with confidence to true differences in critical thinking dispositions, metacognitive weakness, or epistemic laziness, because they may partly reflect group-specific item functioning. For this reason, the between-group comparisons were treated as descriptive information about the samples, whereas the substantive conclusions of the study were restricted to the within-group patterns of associations, which were supported by configural and metric invariance. A more cautious interpretation is that the proposed indirect pattern showed consistency across two distinct student samples.

The findings may also have practical implications for higher education. If GAI use is associated with lower critical thinking dispositions mainly when accompanied by metacognitive weakness and epistemic laziness, educational responses may benefit from moving beyond technical GAI instruction alone (Chan & Hu, 2023). Students may also need support in maintaining reflective engagement during GAI-assisted tasks (Morais et al., 2026). Possible approaches include encouraging students to explain and evaluate how they used GAI and to compare generated responses with alternative sources of information (Adewumi et al., 2025; Lee et al., 2025). Such practices are broadly consistent with evidence suggesting that GAI may support higher-order thinking when learners remain actively involved in reflection and evaluation (Yang et al., 2026; Zhao et al., 2025). From this perspective, GAI literacy initiatives may benefit from helping students engage with GAI while maintaining the cognitive processes that support critical thinking (Yang et al., 2026). At the institutional level, these efforts may be complemented by the responsible governance of GAI in higher education. Possible strategies include transparent institutional policies that specify acceptable forms of GAI use, assessment designs that require students to justify and verify GAI-assisted work, and structured classroom activities in which students critique, correct, and extend GAI outputs. Given that similar indirect patterns were observed in both the Turkish-speaking and English-speaking samples, such strategies may be relevant across different higher education contexts, although their implementation should be adapted to local instructional cultures and resources.

## 5. Conclusions and Limitations

The main objective of the present study was to examine whether metacognitive weakness and epistemic laziness individually and sequentially account for the association between GAI tool usage and critical thinking dispositions during GAI use across two distinct university student samples. This objective was achieved, and the main research question of the study was answered affirmatively. Consistent with the hypotheses, GAI tool usage was indirectly associated with lower critical thinking dispositions during GAI use through metacognitive weakness, through epistemic laziness, and through the sequential pathway linking both constructs, and this pattern was observed in both the Turkish-speaking and English-speaking samples.

These findings suggest that understanding GAI use may require attention not only to how often students use these systems but also to how they engage with them. By showing that metacognitive weakness and epistemic laziness were associated with critical thinking dispositions both individually and sequentially across two student samples, the study provides preliminary support for a process-oriented perspective on GAI-assisted critical thinking. More broadly, the findings suggest that supporting critical thinking in GAI-assisted learning may involve helping students remain engaged in evaluating information and reflecting on their own understanding, and that such support may need to address both self-monitoring and epistemic effort. At the same time, the conclusions of the study are bounded by its design and measurement characteristics, and several limitations should therefore be acknowledged.

First, the cross-sectional design does not allow conclusions regarding causality or temporal ordering. Although the sequential pathway was theoretically specified and supported across both samples, the findings do not indicate that greater GAI use leads to metacognitive weakness, which then results in epistemic laziness and lower critical thinking dispositions. Alternative patterns may also be plausible. For example, students with lower critical thinking dispositions may engage with GAI differently, or students with lower epistemic engagement may rely more on GAI-generated responses. Future longitudinal and experimental research may help clarify whether the proposed sequence remains consistent over time.

Second, all focal variables were measured using self-report instruments. Although self-report measures are useful for examining students’ perceptions during GAI use, they may also reflect common method variance and limited awareness of one’s own cognitive processes. This issue may be particularly relevant for metacognitive weakness and epistemic laziness because students may not always recognize when they are not actively monitoring understanding or evaluating information. Future studies may strengthen this evidence by combining self-report measures with behavioral indicators such as verification behavior and performance-based assessments of critical thinking. Relatedly, the CTAIUS assesses critical thinking dispositions rather than critical thinking skills, and self-assessments of one’s own thinking may be imperfectly calibrated with actual performance. Consequently, the present findings speak to how students perceive and report their engagement with GAI-generated information, and they should not be interpreted as evidence regarding students’ performance-based critical thinking abilities.

In addition, the MWAIUS and ELS capture only the maladaptive pole of their respective constructs. They do not assess adaptive metacognition or active epistemic engagement, which may have restricted the measured range of these constructs. This limitation is particularly relevant to the metacognitive weakness measure. Metacognitive functioning is more appropriately viewed as a continuum ranging from poor monitoring and regulation to highly adaptive functioning, whereas the MWAIUS captures only the deficit end of this continuum. Consequently, the observed associations may not reflect the role of metacognitive functioning across its full range, and the reported coefficients should be interpreted accordingly. In addition, low MWAIUS scores indicate only the relative absence of the measured weaknesses and should not be interpreted as evidence of strong or adaptive metacognitive functioning. A measure spanning the full continuum might therefore yield associations that differ in magnitude or even direction from those observed in the present study. Future research using performance-based critical thinking tasks and bipolar measures of metacognitive functioning would provide a stronger test of the proposed model.

Third, the two samples differed in demographic and recruitment characteristics. The Turkish-speaking sample consisted of undergraduate students from a faculty of education at a public university in Türkiye, whereas the English-speaking sample was recruited through Prolific and included students from more varied educational backgrounds. Although observing a similar indirect pattern across samples may support the broader relevance of the findings, these differences limit strong cross-cultural interpretation. The results should therefore be interpreted as evidence of cross-sample consistency rather than broader cultural generalizability. In addition, scalar invariance was not supported, and any observed group differences should be interpreted descriptively rather than as evidence of equivalent latent differences. Moreover, sample heterogeneity may also have influenced the estimated indirect effects, because differences in age, educational level, and recruitment mode could contribute to differences in the strength of individual paths across the samples. The consistency reported in this study therefore refers to the direction and significance of the indirect associations rather than to the equality of their magnitudes.

Fourth, the structural analyses were conducted using observed composite scores rather than fully latent structural models. This approach was suitable for evaluating the focal indirect associations but did not explicitly account for measurement error. Future studies with larger and more balanced samples may re-examine the proposed model using latent variables and more stringent tests of indirect associations. Relatedly, although a small number of CTAIUS items showed comparatively lower factor loadings, the original 11-item structure was retained. Removing these items did not meaningfully improve model fit, reliability, or convergent validity, while reducing the conceptual breadth of the construct and comparability with the original instrument. The lower-loading items were judged to capture theoretically relevant aspects of critical thinking dispositions in GAI use, and their performance should be re-examined in future validation studies using independent samples.

Fifth, the study assessed general patterns of GAI tool usage without distinguishing between different forms of engagement. The implications of GAI use may vary depending on how students interact with these systems and the educational context in which they are used. Recent evidence suggests that GAI may support higher-order thinking when use remains structured and reflective, whereas less engaged forms of use may be associated with lower autonomy and critical engagement (Yang et al., 2026; Zhao et al., 2025). Future research may benefit from examining more differentiated profiles of GAI use and exploring whether patterns of reflective engagement differ from more convenience-oriented forms of use. In particular, future studies should examine whether educational, creative, problem-solving, and more passive forms of GAI use show different associations with metacognitive weakness, epistemic laziness, and critical thinking dispositions, because the processes examined in this study may be more strongly related to some usage types than to others.

## Figures and Tables

**Table 1:** Participants’ demographic characteristics.

Variable		N	%
Turkish sample			
Gender			
	Women	361	81.9
	Men	80	18.1
GAI use purpose			
	Education	233	52.8
	General knowledge	188	42.6
	Social interaction	20	4.5
Weekly GAI use duration			
	Less than 2 h	322	73.1
	Between 2 and 5 h	83	18.8
	Between 5 and 8 h	24	5.4
	Between 8 and 11 h	8	1.8
	More than 11 h	4	0.9
English-speaking sample			
Gender			
	Women	121	55.5
	Men	97	44.5
Country of residence			
	United States	84	38.5
	United Kingdom	61	28.0
	Canada	57	26.1
	Other	16	7.3
Current education status			
	Bachelor’s student	139	63.8
	Master’s student	46	21.1
	Phd student	16	7.3
	Other (unknown)	17	7.8
GAI use purpose			
	Education	168	77.1
	General knowledge	42	19.3
	Social interaction	8	3.6
Weekly GAI use duration			
	Less than 2 h	54	24.8
	Between 2 and 5 h	78	35.8
	Between 5 and 8 h	51	23.4
	Between 8 and 11 h	20	9.2
	More than 11 h	15	6.9

Note. GAI = generative artificial intelligence.

**Table 2:** Descriptive statistics, reliability variables, and Pearson’s correlations of the study variables.

	ω	α	CR	AVE	1	2	3	4
1. Critical thinking dispositions	0.85, 0.86	0.85, 0.86	0.85, 0.86	0.35, 0.37	**-**			
2. GAI tool usage	0.90, 0.90	0.90, 0.90	0.90, 0.90	0.49, 0.51	−0.16 **, −0.20 ***	-		
3. Metacognitive weakness	0.74, 0.76	0.74, 0.75	0.73, 0.77	0.37, 0.42	−0.38 ***, −0.50 ***	0.33 ***, 0.38 ***	-	
4. Epistemic laziness	0.81, 0.85	0.82, 0.85	0.80, 0.84	0.45, 0.51	−0.51 ***, −0.52 ***	0.40 ***, 0.46 ***	0.58 ***, 0.72 ***	-
5. Age					0.00, 0.01	0.08, 0.23 **	0.05, 0.02	0.00, 0.04
6. Gender					−0.00, −0.18 **	0.00, 0.06	0.10 *, 0.03	0.07, 0.18 **
7. Weekly GAI use					−0.01, −0.06	0.32 ***, 0.59 ***	0.11 *, 0.18 *	0.22 ***, 0.24 ***
Mean score					3.54, 3.93	3.30, 3.20	2.49, 2.24	2.33, 2.19
Standard deviation					0.58, 0.58	0.78, 0.77	0.71, 0.71	0.79, 0.76
Skewness					0.11, −0.82	−0.28, −0.27	0.17, 0.56	0.38, 0.45
Kurtosis					0.15, 2.12	0.22, −0.19	0.11, 0.54	0.10, 0.13

Note. ω = McDonald’s Omega reliability coefficient; CR = composite reliability; AVE = average variance extracted; α = Cronbach’s alpha reliability coefficient; GAI = generative artificial intelligence; Values to the left of the comma belong to the Turkish-speaking group, whereas values to the right belong to the English-speaking group. * *p* < .05, ** *p* < .01, *** *p* < .001.

**Table 3:** CFA model fit indices across the Turkish-speaking and English-speaking samples, along with recommended cut-off values.

Fit Indices	Reference Values	Turkish-Speaking Sample				English-Speaking Sample			
		CTAIUS (N = 441)	AITUS (N = 441)	MWAIUS (N = 441)	ELS (N = 441)	CTAIUS (N = 218)	AITUS (N = 218)	MWAIUS (N = 218)	ELS (N = 218)
χ^2^/df	≤5 (Byrne, 2010)	3.24	3.97	2.14	3.44	2.50	1.88	1.28	1.74
RMSEA	≤0.08 (Hu & Bentler, 1999)	0.07	0.08	0.05	0.07	0.08	0.06	0.04	0.06
SRMR	≤0.08 (Byrne, 2010)	0.05	0.04	0.02	0.02	0.06	0.03	0.03	0.02
CFI	≥0.90 (Hu & Bentler, 1999)	0.93	0.96	0.99	0.99	0.92	0.98	0.99	0.99
GFI	≥0.90 (Hu & Bentler, 1999)	0.95	0.96	0.99	0.99	0.92	0.95	0.99	0.99

Note. CFA = Confirmatory factor analysis; χ^2^ = chi-square statistic; df = degrees of freedom; CFI = comparative fit indices; GFI = goodness of fit index; RMSEA = root mean square error of approximation; CTAIUS = Critical Thinking in AI Usage Scale; AITUS = Artificial Intelligence Tool Usage Scale; MWAIUS = Metacognitive Weakness in AI Use Scale; ELS = Epistemic Laziness Scale.

**Table 4:** Measurement invariance across Turkish- and English-speaking samples.

Scale	Model	χ^2^(df)	CFI	TLI	RMSEA	Comparison	ΔCFI	ΔTLI	ΔRMSEA	Decision
CTAIUS	Configural (M1)	2.867	0.928	0.905	0.053	-	-	-	-	Configural invariance supported
	Metric (M2)	2.782	0.923	0.909	0.052	M2 vs. M1	−0.005	+0.004	−0.001	Metric invariance supported
	Scalar (M3)	3.914	0.872	0.852	0.067	M3 vs. M2	−0.051	−0.057	+0.015	Scalar invariance not supported
AITUS	Configural (M1)	2.922	0.966	0.950	0.054	-	-	-	-	Configural invariance supported
	Metric (M2)	2.994	0.959	0.949	0.055	M2 vs. M1	−0.007	−0.001	+0.001	Metric invariance supported
	Scalar (M3)	4.310	0.930	0.915	0.071	M3 vs. M2	−0.029	−0.034	+0.016	Scalar invariance not supported
MWAIUS	Configural (M1)	1.713	0.993	0.981	0.033	-	-	-	-	Configural invariance supported
	Metric (M2)	1.449	0.993	0.988	0.026	M2 vs. M1	0.000	+0.007	−0.007	Metric invariance supported
	Scalar (M3)	4.977	0.932	0.896	0.078	M3 vs. M2	−0.061	−0.092	+0.052	Scalar invariance not supported
ELS	Configural (M1)	2.593	0.989	0.973	0.049	-	-	-	-	Configural invariance supported
	Metric (M2)	2.065	0.989	0.982	0.040	M2 vs. M1	0.000	+0.009	−0.009	Metric invariance supported
	Scalar (M3)	4.080	0.966	0.948	0.068	M3 vs. M2	−0.023	−0.034	+0.028	Scalar invariance not supported

Note. χ^2^ = chi-square statistic; df = degrees of freedom; CFI = comparative fit indices; TLI = Tucker–Lewis index; RMSEA = root mean square error of approximation; CTAIUS = Critical Thinking in AI Usage Scale; AITUS = Artificial Intelligence Tool Usage Scale; MWAIUS = Metacognitive Weakness in AI Use Scale; ELS = Epistemic Laziness Scale.

**Table 5:** Standardized estimates of total, direct, and indirect effects on critical thinking dispositions for Turkish- and English-speaking samples.

	Effect (S.E.)	
	Turkish-Speaking Sample (N = 441)	English-Speaking Sample (N = 218)
GAI tool use → Critical thinking dispositions (total effect)	−0.18 *** (0.05)	−0.23 *** (0.08)
→ Critical thinking dispositions (direct effect)	0.05 (0.05)	0.02 (0.08)
→ Critical thinking dispositions (total indirect effect)	−0.23 *** (0.03)	−0.26 *** (0.05)
→ Metacognitive weakness → Critical thinking dispositions	−0.05 * (0.02)	−0.11 *** (0.03)
→ Epistemic laziness → Critical thinking dispositions	−0.11 *** (0.02)	−0.07 *** (0.03)
→ Metacognitive weakness → Epistemic laziness → Critical thinking dispositions	−0.08 *** (0.01)	−0.08 *** (0.03)
Metacognitive weakness → Critical thinking dispositions (total effect)	−0.37 *** (0.05)	−0.49 *** (0.07)
→ Critical thinking dispositions (direct effect)	−0.14 ** (0.05)	−0.29 ** (0.09)
→ Epistemic laziness → Critical thinking dispositions (indirect effect)	−0.23 *** (0.03)	−0.20 ** (0.06)
Epistemic laziness → Critical thinking dispositions (direct effect)	−0.46 *** (0.05)	−0.32 ** (0.09)
Weekly GAI use → Critical thinking dispositions (direct effect)	0.09 (0.05)	0.06 (0.07)
Gender → Critical thinking dispositions (direct effect)	0.04 (0.04)	−0.12 * (0.06)
Age → Critical thinking dispositions (direct effect)	0.01 (0.04)	−0.01 (0.06)
USA → Critical thinking dispositions (direct effect)	-	0.00 (0.10)
UK → Critical thinking dispositions (direct effect)	-	0.04 (0.10)
Canada → Critical thinking dispositions (direct effect)	-	−0.13 (0.10)

Note. GAI = generative artificial intelligence. * *p* < .05, ** *p* < .01, *** *p* < .001.

## Data Availability

The data are available on request from the corresponding author.

## Supplementary Materials

The following supporting information can be downloaded at: https://www.mdpi.com/article/10.3390/jintelligence14070147/s1, Table S1: Confirmatory factor loadings for the study measures. The supplementary file provides the rationale for retaining the original 11-item structure of the Critical Thinking in AI Usage Scale (CTAIUS) and presents the standardized confirmatory factor loadings for all study measures in both samples.
