# Supplementary material for: Generative AI Use and Critical Thinking Dispositions in Higher Education: A Cross-Sample Study of the Sequential Role of Metacognitive Weakness and Epistemic Laziness
Source: J Intell. 2026 Jul 13;14(7):147. doi: 10.3390/jintelligence14070147 (PMC13413021; doi:10.3390/jintelligence14070147)
Supplement: Supplementary file 1 [file jintelligence-14-00147-s001.zip › jintelligence-4367167-supplementary.pdf]

Supplementary File S1. Detailed rationale for the refinement of the Critical Thinking in AI Usage Scale (CTAIUS) and factor loadings for the study measures

Rationale for examining the original 11-item structure

The Critical Thinking in AI Usage Scale (CTAIUS) was originally developed and validated as an 11-item unidimensional measure (Yurt & Kuşci, 2026). To examine whether a more parsimonious version would offer psychometric advantages, we explored alternative models based on the removal of items with comparatively lower standardized factor loadings. However, these modifications did not meaningfully improve model fit, reliability, or convergent validity. Moreover, removing items reduced the conceptual coverage of the construct, as the excluded items captured important aspects of reflective and evaluative thinking, such as idea generation and the integration of AI-generated information with one's own reasoning. Because a shortened version provided no clear psychometric benefit while narrowing construct representation and reducing comparability with the original instrument, the full 11-item structure was retained for all analyses. Standardized factor loadings for all study measures are reported in Table S1.

Interpretive note

As shown in Table S1, most CTAIUS items demonstrated adequate standardized factor loadings in both the Turkish-speaking and English-speaking samples, although a small number of items showed comparatively lower loadings, particularly in the English-speaking sample. Nevertheless, excluding these items did not improve model fit, reliability, or convergent validity, while reducing the conceptual breadth of the construct and comparability with the original instrument. Because the scale was analyzed as an observed composite score, retaining the original 11-item structure was considered the most appropriate approach. The

performance of individual items should be re-examined in future validation studies using independent samples.

Table S1. Confirmatory factor loadings for the study measures

Items in both languages	CFA	
	Turkish-speaking sample	English-speaking sample
**Critical Thinking Dispositions (CTAIUS)**		
1. When evaluating information from AI, I try to understand the general framework of the topic. (Yapay zekâdan gelen bilgileri değerlendirirken, konunun genel çerçevesini anlamaya çalışırım.)	.45	.62
2. I try to blend the information I receive from AI with my own ideas. (Yapay zekâdan elde ettiğim bilgileri kendi fikirlerimle bütünleştirmeye özen gösteririm.)	.55	.42
3. I compare information obtained from AI with different sources. (Yapay zekâdan elde ettiğim bilgileri farklı kaynaklarla karşılaştırırım.)	.57	.64
4. Using AI supports my generation of new ideas. (Yapay zekâ kullanımı, yeni fikirler üretmemi destekler.)	.34	.21
5. Even if the information presented by AI contradicts my thoughts, I evaluate it by questioning. (Yapay zekâdan sunulan bilgiler düşüncelerimle çelişse bile, bunları sorgulayarak değerlendiririm.)	.60	.60
6. I try to evaluate the information presented by AI together with different perspectives. (Yapay zekânın sunduğu bilgileri, farklı bakış açılarını dikkate alarak değerlendirmeye çalışırım.)	.71	.73
7. When making decisions using AI, I pay attention to cause-and-effect relationships. (Yapay zekâ destekli kararlar alırken, neden–sonuç ilişkilerine dikkat ederim.)	.72	.58
8. I reevaluate what I learn from AI by comparing it with my own experiences. (Yapay zekâdan öğrendiklerimi kendi deneyimlerimle karşılaştırarak yeniden değerlendiririm.)	.70	.64
9. I take care to question the reliability of information from AI. (Yapay zekâdan gelen bilgilerin güvenilirliğini sorgulamaya özen gösteririm.)	.66	.74
10. When making decisions based on AI recommendations, I carefully evaluate their possible consequences. (Yapay zekâ önerilerine dayalı kararlar alırken, bu kararların olası sonuçlarını dikkatle değerlendiririm.)	.51	.68
11. When working with AI, I take care to develop my own thinking skills. (Yapay zekâ ile çalışırken, kendi düşünme becerilerimi geliştirmeye özen gösteririm.)	.54	.66
**AI Tool Usage (AITUS)**		
1. I utilize generative AI technologies in my daily decision-making processes. (Günlük hayatımda karar verme süreçlerimde AI (Yapay Zeka) teknolojilerinden faydalanırım.)	.70	.67
2. I use generative AI -supported tools in my academic/professional tasks. (Akademik/iş hayatımdaki görevlerimde düzenli olarak AI (Yapay Zeka) destekli araçları kullanırım.)	.71	.64
3. I ask my questions directly to an generative AI assistant instead of using search engines. (Arama motoru kullanmak yerine sorularımı doğrudan bir AI (Yapay Zeka) asistanına sorarım.)	.66	.73
4. I utilize generative AI technologies to create written content. (Yazılı içerik oluşturmak için AI (Yapay Zeka) teknolojilerinden faydalanırım.)	.67	.75

5. I consult generative AI tools to solve complex problems. (Karmaşık problemleri çözmek için AI (Yapay Zeka)araçlarına danışırım.)	.73	.74
6. I consider generative AI recommendations in my decision-making processes. (Karar verme süreçlerimde AI (Yapay Zeka) önerilerini dikkate alırım.)	.75	.75
7. I use generative AI -supported tools to develop creative ideas. (Yenilikçi fikirler geliştirmek için AI destekli araçları kullanırım.)	.72	.75
8. I interact with various generative AI applications at different times of the day. (Günün farklı zamanlarında çeşitli AI uygulamalarıyla etkileşimde bulunurum.)	.68	.65
9. I prefer generative AI technologies for quick access to information. (Bilgiye hızlı erişmek için AI teknolojilerini tercih ederim.)	.68	.73
**Metacognitive Weakness (MWAIUS)**		
1. I do not decide on the approach I will take before using generative AI. (Yapay zekâ kullanmadan önce nasıl bir yol izleyeceğime nadiren karar veririm.)	.52	.61
2. I neglect to check whether I fully understand generative AI outputs when using them. (Yapay zekâ önerilerini kullanırken tam olarak anlayıp anlamadığımı kontrol etmeyi genellikle ihmal ederim.)	.81	.74
3. I focus on obtaining quick results rather than the accuracy of generative AI outputs. (Yapay zekâ önerilerinin doğruluğundan çok, hızlı sonuç almaya odaklanırım.)	.63	.83
4. I do not know which generative AI tool would be more useful in which situation. (Hangi yapay zekâ aracının hangi durumda daha yararlı olacağını genellikle bilmiyorum.)	.40	.29
5. I forget to consider my own strengths and weaknesses when using generative AI. (Yapay zekâ kullanırken kendi güçlü ve zayıf yönlerimi dikkate almayı unuturum.)	.59	.64
**Epistemic Laziness (ELS)**		
1. I neglect to form my own ideas before receiving generative AI recommendations. (Yapay zekânın önerilerini almadan önce kendi fikirlerimi oluşturmayı ihmal ederim.)	.56	.60
2. I do not make much effort to develop my own solutions before consulting generative AI. (Yapay zekâya danışmadan önce kendi çözümümü geliştirmeye pek uğraşmam.)	.67	.64
3. I accept information from generative AI without questioning it. (Yapay zekâdan gelen bilginin doğruluğunu sorgulama zahmetine girmem.)	.75	.77
4. Instead of understanding complex topics in depth, I prefer to try a new generative AI tool. (Karmaşık konuları derinlemesine anlamak yerine, yeni bir yapay zekâ aracı denemeyi tercih ederim.)	.65	.75
5. I avoid expending extra effort to verify the accuracy of generative AI recommendations. (Yapay zekânın önerdiği bilgileri derinlemesine araştırmak için zihinsel çaba göstermeyi gereksiz bulurum.)	.71	.79
